# Transthoracic Cross Clamp versus Endoaortic Balloon Occlusion in Minimally Invasive Mitral Valve Surgery: A Pooled Study with Subgroup Analyses

**DOI:** 10.3390/jcm13174989

**Published:** 2024-08-23

**Authors:** Dimitrios E. Magouliotis, Serge Sicouri, Massimo Baudo, Yoshiyuki Yamashita, Andrew Xanthopoulos, Arian Arjomandi Rad, Thanos Athanasiou, Basel Ramlawi

**Affiliations:** 1 Department of Cardiac Surgery Research, Lankenau Institute for Medical Research, Main Line Health, Wynnewood, PA 19096, USA; sicouris@mlhs.org (S.S.); massimo.baudo@icloud.com (M.B.); yamashitay@mlhs.org (Y.Y.); basel.ramlawi@gmail.com (B.R.); 2 Department of Cardiac Surgery, Lankenau Heart Institute, Main Line Health, Wynnewood, PA 19096, USA; 3 Department of Cardiology, University of Thessaly, 412 23 Larissa, Greece; andrewvxanth@gmail.com; 4 Division of Medical Sciences, University of Oxford, Oxford OX1 2JD, UK; arian.arjomandirad@medsci.ox.ac.uk; 5 Department of Surgery and Cancer, Imperial College London, St Mary’s Hospital, London W2 1NY, UK; t.athanasiou@imperial.ac.uk

**Keywords:** MIMVS, mitral valve surgery, transthoracic clamping, balloon occlusion, TTC, EABO

## Abstract

**Objective**: We assessed the available literature regarding patients undergoing minimally invasive mitral valve surgery (MIMVS) with either transthoracic clamping (TTC) or endoaortic balloon occlusion (EABO). **Methods**: Original research studies that evaluated the perioperative outcomes of TTC versus EABO group were identified from 2000 to 2024. The incidence of all-cause mortality, cerebrovascular accidents (CVA), and aortic dissections were the primary endpoints. The cardiopulmonary bypass (CPB), cross-clamp, and ventilation time, along with the incidence of conversion to sternotomy, re-exploration, new-onset atrial fibrillation (AF), postoperative acute kidney injury (AKI), ICU stay, and LOS were the secondary endpoints. Subgroup analyses were performed regarding the EABO cannulation approach (femoral and aortic) and MIMVS approach (video-assisted and robotic-assisted). Sensitivity analyses were performed with the leave-one-out method and by including risk-adjusted populations. **Results**: Sixteen studies were included in both the qualitative and quantitative syntheses. After pooling data from 6335 patients, both groups demonstrated similar outcomes on all primary and secondary endpoints in the non-adjusted and adjusted total cohort analyses. These outcomes were further validated by the leave-one-out sensitivity analysis. In addition, the aortic cannulation EABO was associated with a lower cross-clamp time, followed by TTC and the femoral cannulation EABO approach. Furthermore, in the video-assisted subgroup analysis, the EABO approach was associated with a higher incidence of CVA, conversion to sternotomy, and longer ICU stay compared to the TTC group. **Conclusions**: The present meta-analysis indicates that both aortic occlusion techniques are safe and feasible in the context of MIMVS. A future well-designed randomized-control trial should further validate the current outcomes.

## 1. Introduction

Surgery remains the gold standard treatment approach for severe mitral valve regurgitation [1]. In fact, the wide adoption of minimally invasive, endoscopic, and robot-assisted techniques in numerous centers is driven by their feasibility and effectiveness, reduced risk of infection, and enhanced patient satisfaction in terms of cosmesis and pain, along with a shorter length of hospital stay [2]. The DeBakey cross-clamp has been the mainstay of aortic occlusion during open cardiac surgery [3]. Nonetheless, aortic occlusion and myocardial protection strategies underwent further adaptations following the increasing adoption of minimally invasive approaches in mitral valve surgery [4]. In the context of minimally invasive mitral valve surgery (MIMVS), the transthoracic clamp (TTC) and the endoaortic balloon occlusion (EABO) approaches have been employed as alternative strategies for aortic occlusion and myocardial protection [5]. TTC incorporates a longer DeBakey-type clamp inserted through the intercostal spaces [5]. On the other hand, EABO employs a transcatheter intraluminal balloon as an alternative strategy for aortic occlusion and myocardial protection [4].

To date, there have been two previous meta-analyses on this topic available in the literature [5,6]. The first meta-analysis [6] was associated with limitations related to the lack of a sensitivity analysis regarding the cannulation site in the EABO approach. Moreover, both meta-analyses were associated with two additional limitations: (1) they did not include larger multicentric studies with adjusted outcomes published in the five-year interval since the last meta-analysis [5], (2) they did not perform sensitivity analyses (a) with risk-adjusted populations and (b) using the leave-one-out method, and (3) they did not perform subgroup analyses regarding the MIMVS setting (video-assisted or robotic-assisted). The first point is important since there are no available randomized control trials and most of the previous studies were small with most surgeons exclusively using one technique or the other, especially given the steep learning curve of the EABO approach, thus posing a certain bias. The second point (sensitivity analysis) is necessary to provide the best up-to-date level of evidence given the increasing popularity of robot-assisted mitral valve surgery. Aiming to address these issues, we performed an updated meta-analysis comparing TTC and EABO as two alternative strategies for aortic occlusion and myocardial protection in the setting of minimally invasive and robot-assisted mitral valve surgery.

## 2. Materials and Methods

### 2.1. Literature Search and Articles Selection Strategy

We conducted the present study in accordance with the protocol agreed by all participating authors following the Preferred Reporting Items for Systematic Reviews and Meta-Analyses (PRISMA) [7]. The PRISMA Checklist 2020 is demonstrated in Table S1. A thorough literature search was performed in three databases: PubMed/Medline, Scopus/ELSEVIER, and the Cochrane Central Register of Controlled Studies (CENTRAL) (the last search was performed on 24 June 2024). The following terms were employed in every possible combination: “transthoracic clamp”, “cross-clamp”, “ttc”, “aortic balloon”, “eabo”, “aortic occlusion”, “mitral valve replacement”, “mitral valve surgery”, “mitral valve repair”, “mvr”, “mimvs”, and “minimally invasive mitral valve surgery”. Inclusion criteria were (1) original reports written in English, (2) with ≥ 10 patients, (3) published between 2000 and 2024, (4) conducted on human subjects, and (5) reporting comparative outcomes of patients undergoing MIMVS (including robot-assisted) with the employment of either the TTC or EABO approach for aortic occlusion. All duplicate articles were excluded. We also reviewed the reference lists of all included articles for additional studies. Two authors (DEM, SS) worked independently and extracted data from the included studies. Any potential discrepancies between the two investigators were further discussed with the senior author (BR) to include only articles that best matched the criteria until consensus was reached.

### 2.2. Data Extraction and Endpoints

Data were extracted from each eligible study relative to the demographics (number of patients, gender, age, type of TTC, type of EABO, previous cerebrovascular events), along with the incidence of all-cause mortality, cerebrovascular accident (CVA), aortic dissection, aortic cross-clamp and cardiopulmonary bypass (CPB) time, the incidence of conversion to sternotomy, re-exploration, new onset atrial fibrillation (AF), postoperative acute kidney injury (AKI), ventilation time, intensive care unit (ICU) stay, and length of hospital stay (LOS). The incidence of all-cause mortality, CVA, and aortic dissection were the primary endpoints. Aortic cross-clamp and CPB time, along with the incidence of conversion to sternotomy, re-exploration, new onset AF, postoperative AKI, ventilation time, ICU stay, and LOS were the secondary endpoints.

### 2.3. Sensitivity Analysis on Primary Endpoints

Aiming to validate our findings, we conducted further sensitivity analyses regarding both the primary and secondary endpoints. First, we performed subgroup analyses using the following subgroups: (1) EABO with aortic cannulation, (2) EABO with femoral cannulation, (3) video-assisted approach, (4) robotic-assisted approach, and (5) only studies with risk-adjusted patient groups. Second, we conducted further sensitivity analyses by employing the leave-one-out method. The leave-one-out method involves conducting separate meta-analyses on each subset of the studies remaining after leaving out exactly one study.

### 2.4. Quality and Publication Bias Assessment

We evaluated the non-randomized controlled trials (RCTs) for their quality using the Newcastle–Ottawa Quality Assessment Scale (NOS) [8] as an assessment tool. The scale’s range varies from zero to nine stars. Studies with a score equal to or higher than five were considered to have adequate methodological quality and were finally included. All studies with a score lower than five stars were excluded. The Risk of Bias in Non-Randomized Studies of Interventions tool (ROBINS-I) was also employed to assess the risk of bias of the included studies [9]. No RCTs were included in the present meta-analysis. Two reviewers (DEM, SS) rated the studies in an independent manner, and a final decision was reached by consensus. The risk of publication bias was evaluated by visual inspection of the funnel plots.

### 2.5. Statistical Analysis

The odds ratio (ORs) and 95% confidence interval (95% CI) were estimated for the categorical outcomes using the random-effects model (Mantel–Haenszel statistical method). OR < 1 denoted an outcome that was more frequent in the EABO group. The weighted mean difference (WMD) with its 95% CI was calculated for the continuous outcomes using the random-effects (inverse variance statistical method) models. In cases where the WMD was lower than zero, values in the EABO group were higher. We chose the random-effects model since we did not expect that all included studies would share a common effect size. Inter-study heterogeneity was assessed through the Cochran *Q* statistic and by estimating *I*^2^ [10]. Forest plots were also produced regarding the variables that were analyzed. We employed the Cochrane Collaboration Review Manager version 5.4.1 to perform all of the analyses.

## 3. Results

### 3.1. Search Strategy and Patient Demographics

**Figure 1:**
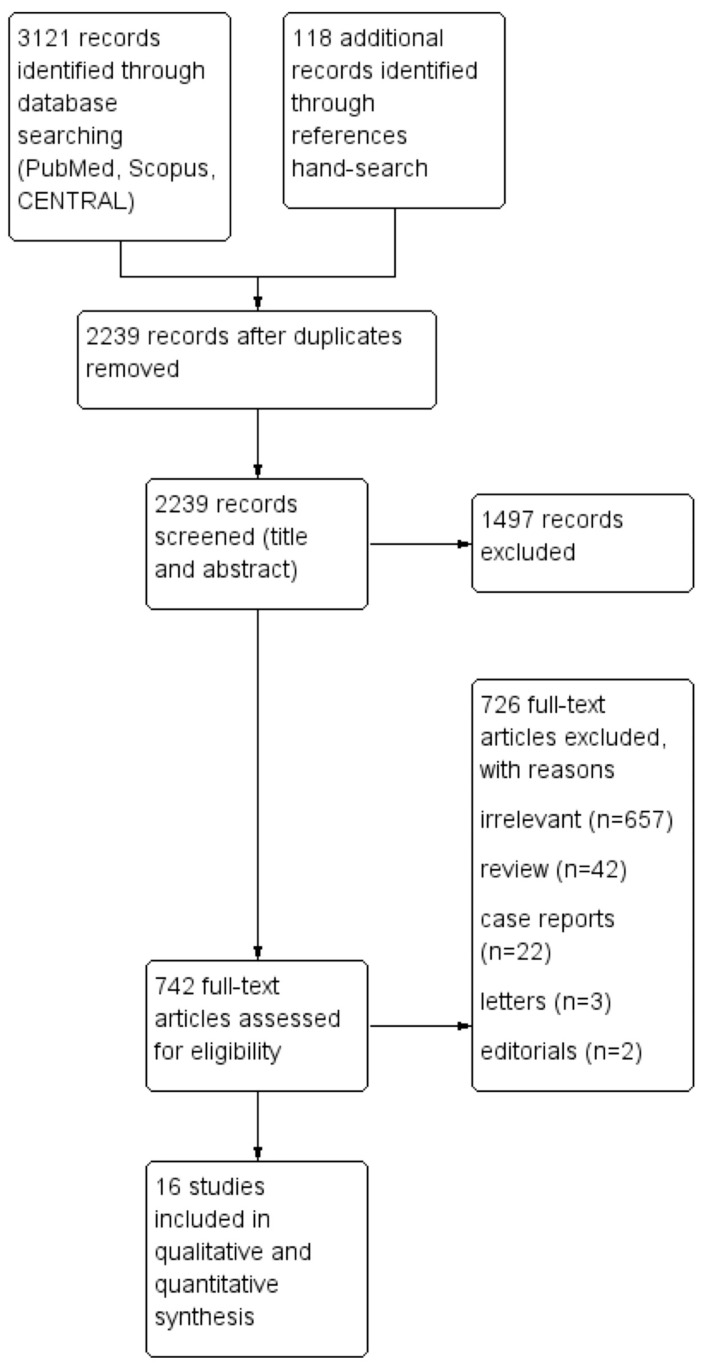
Trial flow of the current meta-analysis.

**Figure 2:**
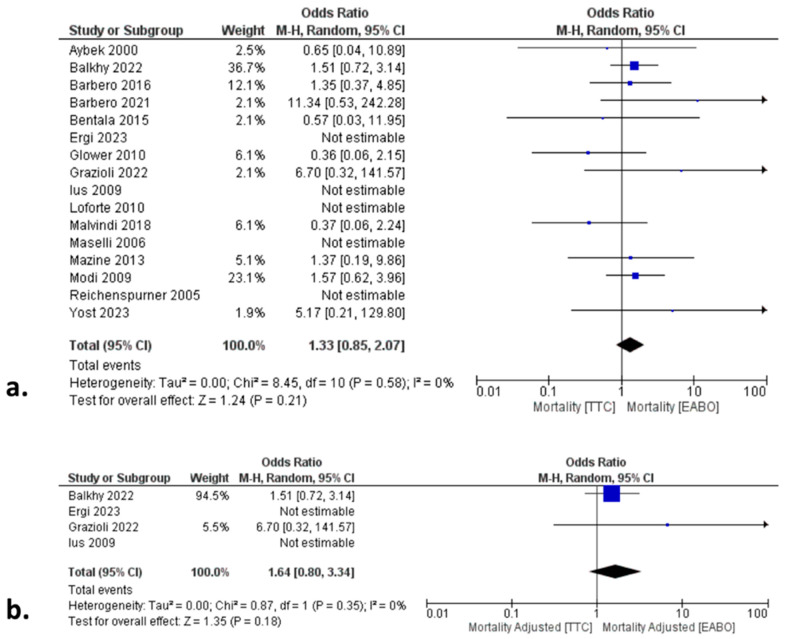
Forest plots regarding all-cause mortality in the (**a**) non-adjusted and (**b**) adjusted total cohort. There were no significant differences between the transthoracic clamping (TTC) and endoaortic balloon occlusion (EABO) groups [11,12,13,14,15,16,17,18,19,20,21,22,23,24,25,26].

The search strategy is demonstrated in the flow diagram in Figure 1 and the PRISMA Checklist 2020 (Supplementary Materials). The characteristics of the included studies are summarized in Table 1. Among the 3239 articles in PubMed/Medline, Scopus/Elsevier, and CENTRAL that were originally identified, sixteen studies [11,12,13,14,15,16,17,18,19,20,21,22,23,24,25,26] were included in the qualitative and quantitative syntheses. The level of agreement between the two reviewers was “almost perfect” (kappa = 0.91; 95% CI: 0.81, 1.00). Figure 2a,b shows the qualitative assessment with the ROBINS-I tool. The main concerns posed by the authors were related to biases due to the selection of participants and performance. The study design was prospective in four studies [14,16,22,24], and retrospective in twelve studies [11,12,13,15,17,18,19,20,21,23,25,26]. PSM was performed in four studies [12,16,18,19]. Three studies [20,21,22] were retrospective using a prospectively collected database. No RCTs were included in the current meta-analysis. The included studies were conducted in Germany [11,24,25], the USA [12,16,17,26], Italy [13,14,19,20,21,22], the Netherlands [15], Canada [23], and one was multinational [18]. The studies were published between 2000 and 2023. The total sample size was 6335 patients (TTC: 3271; EABO: 3064). The ratio of mitral valve repair (MVR) operations ranged from 9% to 73% with significant heterogeneity among studies. The comparison of the two groups in terms of the baseline characteristics is demonstrated in Table 2. The TTC and EABO groups had similar baseline characteristics, except for the previous cardiac surgery variable, with more redo cases incorporated into the EABO group (OR: 0.45; 95% CI: 0.22–0.91; *p* = 0.03). The primary and secondary endpoints, along with the sensitivity subgroup analyses, are demonstrated cumulatively in Table 3.

### 3.2. Primary Endpoints: All-Cause Mortality, CVA, and Aortic Dissection

In the total cohort analysis, there was no significant difference between the two groups in terms of all-cause mortality (HR: 1.33; 95% CI:0.85, 2.07; *p* = 0.21) (Figure 2a), incidence of CVA (OR: 0.68; 95% CI: 0.44, 1.04; *p* = 0.07), and aortic dissection (OR: 0.51; 95% CI: 0.20, 1.33; *p* = 0.17) (Table 3).

### 3.3. Secondary Endpoints

In the total cohort analysis, both groups demonstrated similar CPB (OR: −1.68; 95% CI: −8.21, 4.85; *p* = 0.61), cross-clamp (OR: −3.27; 95% CI: −7.61, 1.07; *p* = 0.14), and ventilation (OR: −0.03; 95% CI: −0.58, 0.52; *p* = 0.92) time. In addition, there was no significant difference between the two groups regarding the incidence of conversion to sternotomy (OR: 0.51; 95% CI: 0.19, 1.39; *p* = 0.19), re-exploration (OR: 0.90; 95% CI: 0.64, 1.28; *p* = 0.57), new-onset AF (OR: 0.86; 95% CI: 0.61, 1.21; *p* = 0.37), and postoperative AKI (OR: 1.22; 95% CI: 0.91, 1.65; *p* = 0.19). Finally, both groups were similar regarding ICU stay (OR: −0.27; 95% CI: −0.72, 0.19; *p* = 0.25) and LOS (OR: 0.30; 95% CI: −0.60, 1.21; *p* = 0.51).

### 3.4. Subgroup and Sensitivity Analyses

To further validate our outcomes, we performed subgroup analyses comparing TTC vs. EABO in patients (a) with femoral cannulation EABO, (b) aortic cannulation EABO, (c) undergoing video-assisted, and (d) robotic-assisted MIMVS. In the femoral EABO subgroup, all outcomes were similar to the TTC group, except for the aortic cross-clamp time, which was higher in the EABO group. In contrast, the aortic EABO subgroup demonstrated significantly lower cross-clamp time compared to the TTC group. Consequently, the aortic cannulation EABO approach was associated with the shortest cross-clamp time of all three subgroups. In the video-assisted subgroup analysis, EABO was associated with a higher incidence of CVA, conversion to sternotomy, and longer ICU stay compared to the TTC group.

Moreover, the validity of the total cohort analysis outcomes was further affirmed by the risk-adjusted subgroup analyses, in which patients were matched with the baseline characteristics to minimize the risk of bias related to cofounders. In fact, the outcomes of this subgroup analysis were similar to the total cohort analysis outcomes, with no difference between the two groups in any of the primary or secondary endpoints (Figure 2b, Table 3). Finally, no difference was found when we applied the leave-one out sensitivity analysis method, thus further supporting the validity of our outcomes.

### 3.5. Quality and Publication Bias Assessment

**Figure 3:**
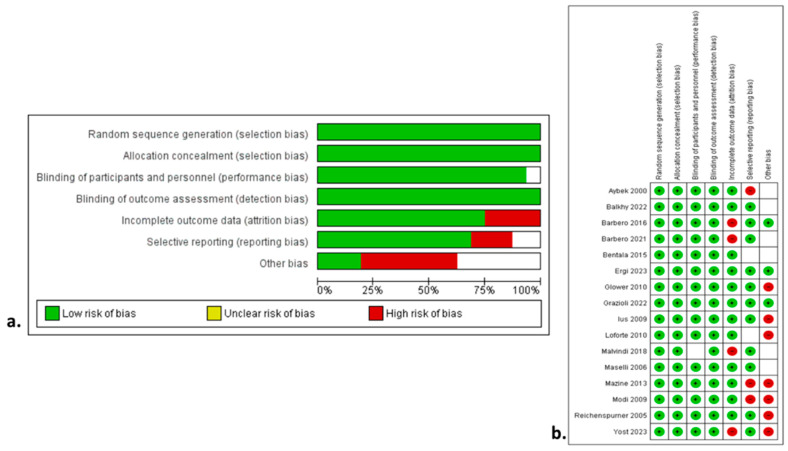
Risk of Bias in Non-Randomized Studies of Interventions with (**a**) summary plot and (**b**) traffic lights [11,12,13,14,15,16,17,18,19,20,21,22,23,24,25,26].

The quality evaluation according to the Newcastle–Ottawa Scale for all studies is shown in Table 1. Figure 3 demonstrates the qualitative assessment of the studies according to the ROBINS-I tool. Figure 3a,b shows the qualitative assessment with the ROBINS-I tool. The authors’ main concerns were mainly related to biases associated with the outcome data and selective reporting. The primary endpoints were associated with low heterogeneity. Most of the secondary endpoints were related to low heterogeneity. In contrast, the CPB and cross-clamp time, along with the incidence of conversion to sternotomy, ICU stay, and LOS, were associated with high heterogeneity. The main factors affecting and increasing heterogeneity in these variables were the level of expertise, the volume of cases, the differences in operation setting, and aortic occlusion devices, along with the differences in the perioperative pathway protocols among different institutions. The funnel plots (Figure S1) seemed asymmetrical, with studies being absent from either the top or bottom of the graph, thus suggesting the presence of certain publication bias. The relatively small number of included studies was the main reason for the reported asymmetry.

## 4. Discussion

The current meta-analysis identified sixteen articles comparing the TTC versus EABO as two alternative methods of aortic occlusion for minimally invasive mitral valve surgery and incorporated 6335 patients. According to our total cohort analysis, TTC and EABO demonstrated comparable outcomes with regard to the primary and secondary outcomes. Although a previous meta-analysis [5] was conducted in 2019 (study period until December 2018), numerous newer studies have been published with important characteristics (PSM study design in three of them [12,16,18] and robotic-assisted MIMVS in two studies [16,26]), and the sensitivity analyses were limited. Given the lack of a randomized trial, the present meta-analysis provides the best currently available level of evidence on this topic.

All included studies reported postoperative all-cause mortality. According to the whole cohort analysis and all related sensitivity analyses, both techniques were associated with a similar all-cause mortality rate. This was an expected outcome given the growing evidence, suggesting that baseline characteristics and CPB time, rather than aortic clamping technique, are predictors of mortality [13,24]. In fact, in the present study, we tried to limit the impact of potential cofounders by assessing the similarity of the baseline characteristics in the total cohort and by performing a PSM sensitivity analysis. Given the low heterogeneity, similarity, and replicability of these outcomes in all sensitivity analyses, we suggest that both techniques are equally safe in terms of all-cause mortality and that survival is not influenced by the aortic occlusion technique.

Fifteen studies reported outcomes on postoperative CVA. The overall cohort analysis showed no difference between the two groups in the risk of CVA with either technique. In addition, the incidence of CVA was similar between the TTC and EABO in either the femoral or the aortic cannulation EABO subgroup. However, in the video-assisted MIMVS subgroup analysis, the incidence of CVA was higher in the EABO cohort. A potential mechanism is the increased risk of embolus derived from the aortic wall of patients with severe atheromatous disease and porcelain aorta during the manipulation of the balloon catheter, and the inflation–deflation–reinflation circles that may occur in cases of balloon migration. Overall, both the TTC and EABO are associated with a similarly low risk of CVA; however, EABO (aortic) seems the least risk prone for this outcome. Nonetheless, the PSM and leave-one-out sensitivity analyses confirmed the equal outcomes demonstrated by the total cohort analysis. Finally, there was zero heterogeneity in all analyses regarding CVA incidence.

Seventeen studies reported CPB time and sixteen studies reported aortic cross-clamp time. There was no difference between the two groups in terms of CPB and cross-clamp time in the total cohort, PSM, and video-assisted approach analyses. Nonetheless, there was high heterogeneity among the included studies, probably attributed to differences in terms of the level of expertise, the point of standing in the learning curve, the volume of cases, the operation setting, the cross-clamp devices, and the perioperative pathway protocols among different institutions. Outcomes were different in the cannulation approach subgroup analyses. However, there was no difference regarding the CPB time in all analyses, and the cross-clamp time was higher in the femoral EABO and lower in the aortic EABO group compared to the TTC group. These results are consistent with the previous meta-analysis [5] regarding cross-clamp time, but differ with respect to CPB time. The main reasons for this difference from the previous meta-analysis were the inclusion of six newer studies with a larger number of patients included as well as surgeons more experienced in MIMVS. However, the difference between the femoral cannulation approach EABO and TTC technique remains, mainly due to the more straightforward nature and shorter learning curve of the TTC occlusion maneuver [27].

Fifteen studies were included in the aortic dissection assessment. According to the total and PSM analyses, both techniques were associated with a similar incidence of aortic dissections. This finding is in contrast with the previous meta-analysis that reported a higher incidence of aortic dissection for the EABO group. In addition, the cannulation approach (femoral or aortic) did not affect our outcomes. There is evidence demonstrating the correlation between the learning curve and the incidence of iatrogenic aortic dissections [28]. Because we included newer studies with larger patient volumes, the impact of learning was limited, and the outcomes were similar between the two groups. Furthermore, according to the total cohort and PSM analyses, there was no difference between the two groups regarding the perioperative morbidity.

The limitations of the current meta-analysis are relevant to the limitations posed by the included studies. No RCTs were included. Although most studies were retrospective in nature, seven of them provided either risk-adjusted/PSM analyses or used prospectively collected data. In addition, the included studies were related to potential biases regarding the outcome data and selective reporting. Moreover, differences among institutions in selection criteria, surgeon expertise, different occlusion devices, and perioperative management pose certain limitations.

On the other hand, the present study was associated with certain strengths such as (1) the clear data-extraction protocol, (2) the well-specified inclusion/exclusion criteria, (3) the literature search performed in three different databases, (4) the quality assessment of the included studies, (5) the detailed presentation of the results of data-extraction and analyses, (6) the significantly larger patient sample compared to the previous meta-analyses, (7) the groups were similar in almost all baseline characteristics, and (8) the thorough sensitivity and subgroups analyses performed.

## 5. Conclusions

In the context of patients undergoing MIMVS, aortic occlusion with either the TTC or EABO approach is similarly safe and feasible. There was no difference between the two groups regarding the primary endpoints (all-cause mortality, CVA, aortic dissections) between the two groups in the non-adjusted and adjusted total cohort analyses. Furthermore, the aortic cannulation EABO approach was associated with the shortest cross-clamp time. The current study represents the best currently available level of evidence on the topic and should be further supported by a well-designed future RCT.

## Figures and Tables

**Table 1:** Baseline characteristics and the quality assessment according to the Newcastle–Ottawa Scale (NOS) of the studies finally included in the meta-analysis.

Study ID, Year	Study Design	Patients, n TTC/EABO	Age, Mean ± SD TTC/EABO	Female Sex, % TTC/EABO	LVEF, Mean ± SD, TTC/EABO	NYHA Class 3/4, % TTC/EABO	Previous CVA, % TTC/EABO	Previous CS, % TTC/EABO	Type of TTC	Type of EABO Cannulation Approach	MVR:MVRe, % TTC/EABO	NOS
Aybek, 2000 [11]	R	35/23	56 ± 13/58 ± 16	46/52	61 ± 11/66 ± 13	3 ± 1/3 ± 1	3/0	N/A	Chitwood	Heartport Endoaortic Clamp	37:63/26:74	5
Balkhy, 2022 [12]	R-STS-A	1163/1163	62 ± 12/62 ± 12	36/36	EF < 30: 1/1	23/23	8/8	6/5	N/A	Intraclude	14:86/14:86	8
Barbero, 2016 [13]	R	150/301	67 ± 12/P:61 ± 14 C: 69 ± 9	43/P: 52 C: 24	61 ± 10/P: 59 ± 11 C: 57 ± 13	N/A	11/P: 6 C: 10	6/P: 32 C: 25	Chitwood	Intraclude	38:62/40:60	6
Barbero, 2021 [14]	P	37/80	62 ± 9/55 ± 12	30/35	63 ± 8/62 ± 7	N/A	N/A	3/10	Chitwood	Intraclude	10:90/21:79	6
Bentala, 2015 [15]	R	57/164	62 (57–73)/66 (60–74)	44/44	EF < 30: 9/4	NYHA III: 54/52	7/2	N/A	Chitwood	Intraclude	16:84/9:91	6
Ergi, 2023 [16]	P-A	168/56	65 (56–70)/66 (55–72)	32/27	63 (59–65)/61.5 (59–65)	17/17	0/0	N/A	Chitwood	IntraClude	N/A	8
Glower, 2010 [17]	R	436/235	59 ± 13/58 ± 14	53/59	51 ± 12/53 ± 10	72/56	N/A	20/14	Cosgrove	Intraclude	22:78/33:67	6
Grazioli, 2022 [18]	R-A	78/102	60 ± 14/6 ± 12	35/49	58 ± 6/56 ± 8	31/48	N/A	N/A	Chitwood, Cygnet	Intraclude	14:86/23:77	7
Ius, 2009 [19]	R-A	95/32	62 ± 11/63 ± 9	49/59	65 ± 8/64 ± 8	26/22	N/A	N/A	Cygnet, Portaclamp, Chitwood	Intraclude	23:77/41:59	7
Loforte, 2010 [20]	R *	93/45	59 ± 8/58 ± 11	73/78	60 ± 10/58 ± 9	40/40	N/A	0/0	Cygnet	Intraclude	77:23/73:27	6
Malvindi, 2018 [21]	R *	165/93	63 ± 13/56 ± 15	47/51	55 ± 7/55 ± 9	N/A	N/A	N/A	N/A	N/A	30:70/42:58	6
Maselli, 2006 [22]	P	16/20	55 ± 5/57 ± 6	63/70	N/A	N/A	N/A	N/A	Chitwood	Intraclude	38:62/45:55	6
Mazine, 2013 [23]	R *	103/140	62 ± 11/55 ± 2	39/40	61 ± 9/61 ± 8	32/31	6/6	3/7	Chitwood	N/A	13:87/20:80	6
Modi, 2009 [24]	P	573/479	61 ± 14	51	N/A	N/A	N/A	7/15	N/A	N/A	20:80/15:85	6
Reichenspurner, 2005 [25]	R	60/60	62 ± 11	71	56 ± 16	N/A	N/A	N/A	Chitwood	Intraclude	33:67	5
Yost, 2023 [26]	R	42/71	62 (56–69)/65 (56–72)	40/30	N/A	N/A	7/11	N/A	Chitwood	IntraClude	2:98/9:91	6

Abbreviations: TTC = transthoracic clamping; EABO = endoaortic balloon occlusion; R = retrospective; P = prospective; A = adjusted; N/A = not available; LVEF = left ventricle ejection fraction; NYHA = New York Heart Association; CVA = cerebrovascular accidents; CS = cardiac surgery; MVR = mitral valve replacement; MVRe = mitral valve repair; SD = standard deviation; Chitwood = Chitwood clamp (Scanlan International, Inc., Minneapolis, MN, USA); Cygnet = Cygnet (Novare Surgical Systems Inc., Cupertino, CA, USA); Portaclamp = Portaclamp (Cardio Life Research SA, Louvain-la-Neuve, Belgium); Cosgrove = Cosgrove Flexible Clamp (Cardinal Health V, Edwards Lifesciences Corporation, Irvine, CA, USA); Intraclude = Intraclude (Edwards Lifesciences, Irvine, CA, USA); Heartport = Heartport Endoaortic Clamp (Heartport, Redwood City, CA, USA). * Retrospective analysis of a prospectively collected database.

**Table 2:** Comparison of baseline characteristics.

Baseline Characteristics	Arms	OR *	95% CI	*p*-Value	Heterogeneity	
					*I* ^2^	*p*-Value
Age	14	0.21	−1.5, 1.93	0.81	90%	<0.01
Female ratio	14	0.92	0.82, 0.73	0.15	0%	0.82
LVEF	10	0.55	−0.49, 1.59	0.30	63%	<0.01
NYHA III/IV	8	1.08	0.80, 1.47	0.06	66%	<0.01
Previous CVA	7	1.04	0.79, 1.36	0.80	0%	0.57
Previous CS	8	0.45	0.22, 0.91	0.03	88%	<0.01
MVR rate	15	0.83	0.65, 1.05	0.12	55%	<0.01

Abbreviations: LVEF = left ventricle ejection fraction; NYHA = New York Heart Association; CVA = cerebrovascular accidents; CS = cardiac surgery; MVR = mitral valve replacement; OR = odds ratio; 95% CI = 95% confidence intervals. * Mantel–Haenszel (M–H) method was employed for categorical variables and inverse variance (IV) for continuous variables.

**Table 3:** Summary of primary and secondary endpoints in the total cohort and subgroup analyses.

Endpoints	Arms	OR *	95% CI	*p*-Value	Heterogeneity	
					*I* ^2^	*p*-Value
Total cohort						
All-cause mortality	16	1.33	0.85, 2.07	0.21	0%	0.58
CPB time	17	−1.68	−8.21, 4.85	0.61	95%	<0.01
Aortic cross-clamp time	16	−3.27	−7.61, 1.07	0.14	92%	<0.01
Conversion to sternotomy	14	0.51	0.19, 1.39	0.19	65%	<0.01
Aortic dissection	15	0.51	0.20, 1.33	0.17	0%	0.50
CVA	15	0.68	0.44, 1.04	0.07	0%	0.59
Re-exploration	14	0.90	0.64, 1.28	0.57	0%	0.61
Ventilation	8	−0.03	−0.58, 0.52	0.92	0%	0.71
New onset AF	10	0.86	0.61, 1.21	0.37	54%	0.03
AKI	11	1.22	0.91, 1.65	0.19	0%	0.85
ICU stay	10	−0.27	−0.72, 0.19	0.25	97%	<0.01
LOS	15	−0.20	−0.99, 0.58	0.61	99%	<0.01
Femoral cannulation EABO						
All-cause mortality	14	1.44	0.91, 2.28	0.12	0%	0.71
CPB time	14	−3.78	−9.84, 2.28	0.22	94%	<0.01
Aortic cross-clamp time	13	−5.60	−10.47, −0.73	0.02	93%	<0.01
Conversion to sternotomy	14	0.52	0.19, 1.40	0.20	65%	<0.01
Aortic dissection	14	0.51	0.20, 1.33	0.17	0%	0.50
CVA	15	0.66	0.43, 1.02	0.06	0%	0.65
Re-exploration	13	0.87	0.61, 1.24	0.45	0%	0.59
Ventilation	7	−0.04	−0.59, 0.51	0.89	0%	0.64
New onset AF	9	1.12	0.93, 1.35	0.22	0%	0.44
AKI	10	1.27	0.93, 1.72	0.13	0%	0.88
ICU stay	9	−0.30	−0.78, 0.18	0.22	98%	<0.01
LOS	13	−0.20	−1.17, 0.77	0.69	99%	<0.01
Aortic cannulation EABO						
All-cause mortality	2	1.51	0.72, 3.14	0.21	N/A	−
CPB time	3	10.07	−35.55, 55.49	0.66	98%	<0.01
Aortic cross-clamp time	3	7.89	3.65, 12.12	<0.01	0%	0.42
Conversion to sternotomy	2	0.14	0.01, 3.44	0.23	N/A	−
Aortic dissection	2	N/E	−	−	−	−
CVA	2	3.01	0.15, 59.20	0.47	N/A	−
Re-exploration	2	1.60	0.51, 4.97	0.42	0%	0.75
Ventilation	1	2.60	−6.73, 11.93	0.58	N/A	−
New onset AF	1	0.45	0.27, 0.76	<0.01	N/A	−
AKI	2	0.73	0.25, 2.08	0.13	0%	0.88
ICU stay	1	0.10	−0.70, 0.90	0.81	N/A	−
LOS	2	0.00	−0.10, 0.10	0.99	0%	0.40
Video-assisted approach						
All-cause mortality	12	1.18	0.67, 2.08	0.57	0%	0.48
CPB time	13	−4.85	−14.51, 4.80	0.32	94%	<0.01
Aortic cross-clamp time	12	−5.41	−11.54, 0.72	0.08	89%	<0.01
Conversion to sternotomy	10	0.31	0.16, 0.61	<0.01	0%	0.69
Aortic dissection	11	0.39	0.14, 1.13	0.08	0%	0.45
CVA	11	0.55	0.31, 0.98	0.04	0%	0.52
Re-exploration	12	0.87	0.61, 1.23	0.43	0%	0.43
Ventilation	8	−0.03	−0.58, 0.52	0.92	0%	0.71
New onset AF	8	0.77	0.52, 1.14	0.19	37%	0.14
AKI	10	1.08	0.59, 1.97	0.81	0%	0.79
ICU stay	8	−0.07	−0.09, −0.05	<0.01	0%	0.88
LOS	12	−0.40	−1.36, 0.57	0.42	99%	<0.01
Robotic-assisted approach						
All-cause mortality	2	5.17	0.21, 129.80	0.32	N/A	−
CPB time	2	13.68	7.31, 20.05	<0.01	94%	<0.01
Aortic cross-clamp time	2	4.46	−4.36, 13.28	0.32	98%	<0.01
Conversion to sternotomy	2	1.71	0.10, 280.03	0.71	N/A	−
Aortic dissection	2	1.01	0.04, 25.20	0.99	N/A	−
CVA	2	0.55	0.02, 13.88	0.72	N/A	−
Re-exploration	2	5.38	0.54, 53.54	0.15	N/A	−
Ventilation	0	−	−	−	−	−
New onset AF	1	1.38	0.49, 3.86	0.54	N/A	−
AKI	0	−	−	−	−	−
ICU stay	0	−	−	−	−	−
LOS	1	0.00	−0.14, 0.14	1.00	N/A	−
Risk-Adjusted Total Cohort						
All-cause mortality	4	1.64	0.80, 3.34	0.18	0%	0.35
CPB time	4	0.51	−9.03, 10.06	0.92	89%	<0.01
Aortic cross-clamp time	4	−3.84	−9.16, 1.49	0.16	81%	<0.01
Conversion to sternotomy	4	0.51	0.05, 5.54	0.58	84%	<0.01
Aortic dissection	4	0.90	0.14, 5.93	0.91	29%	0.24
CVA	4	0.71	0.35, 1.44	0.34	7%	0.34
Re-exploration	3	1.03	0.04, 25.96	0.98	N/A	−
Ventilation	2	−5.77	−19.75, 8.21	0.42	58%	0.12
New onset AF	4	1.18	0.97, 1.44	0.10	0%	0.50
AKI	4	1.33	0.96, 1.84	0.09	0%	0.39
ICU stay	4	−0.50	−1.28, 0.28	0.21	99%	<0.01
LOS	4	0.30	−0.60, 1.21	0.51	95%	<0.01

Abbreviations: EABO = endoaortic balloon occlusion; CVA = cerebrovascular accidents; AF = atrial fibrillation; CPB = cardiopulmonary bypass; AKI = acute kidney injury; ICU = intensive care unit; LOS = length of hospital stay; OR = odds ratio; 95% CI = 95% confidence intervals; N/A = Not available. * Mantel–Haenszel (M–H) method was employed for categorical variables and inverse variance (IV) for continuous variables.

## Data Availability

The data that support the findings of this study are available from the corresponding author, upon reasonable request.

## Supplementary Materials

The following supporting information can be downloaded at: https://www.mdpi.com/article/10.3390/jcm13174989/s1, Figure S1: Funnel plots describing the publication bias regarding (a) mean operative time (MOT), (b) intraoperative blood loss, (c) length of stay (LOS), (d) complications; Table S1: PRISMA 2020 Checklist.
