# Supplementary material for: Transthoracic Cross Clamp versus Endoaortic Balloon Occlusion in Minimally Invasive Mitral Valve Surgery: A Pooled Study with Subgroup Analyses
Source: J Clin Med. 2024 Aug 23;13(17):4989. doi: 10.3390/jcm13174989 (PMC11396219; doi:10.3390/jcm13174989)
Supplement: Supplementary file 1 [file jcm-13-04989-s001.zip › Figure S1.pdf]

Figure S1. Funnel plots describing the publication bias regarding (a) mean operative time (MOT), (b) intraoperative blood loss, (c) length of stay (LOS), (d) complications

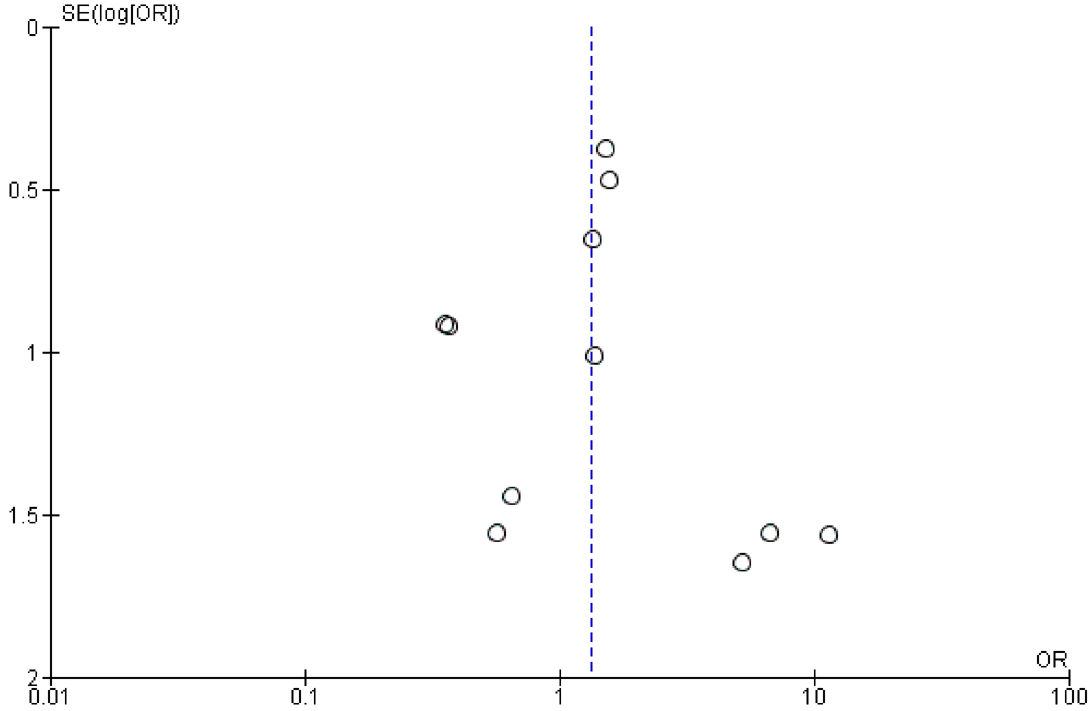

a. Funnel plot regarding Mortality.

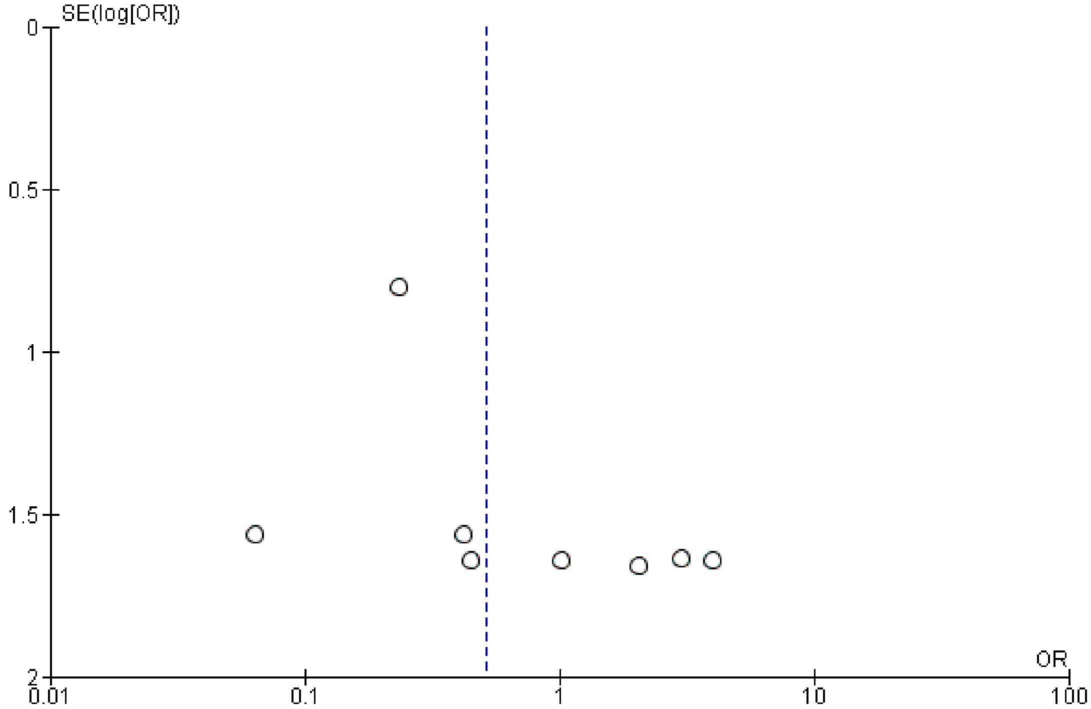

b. Funnel plot regarding aortic dissection.

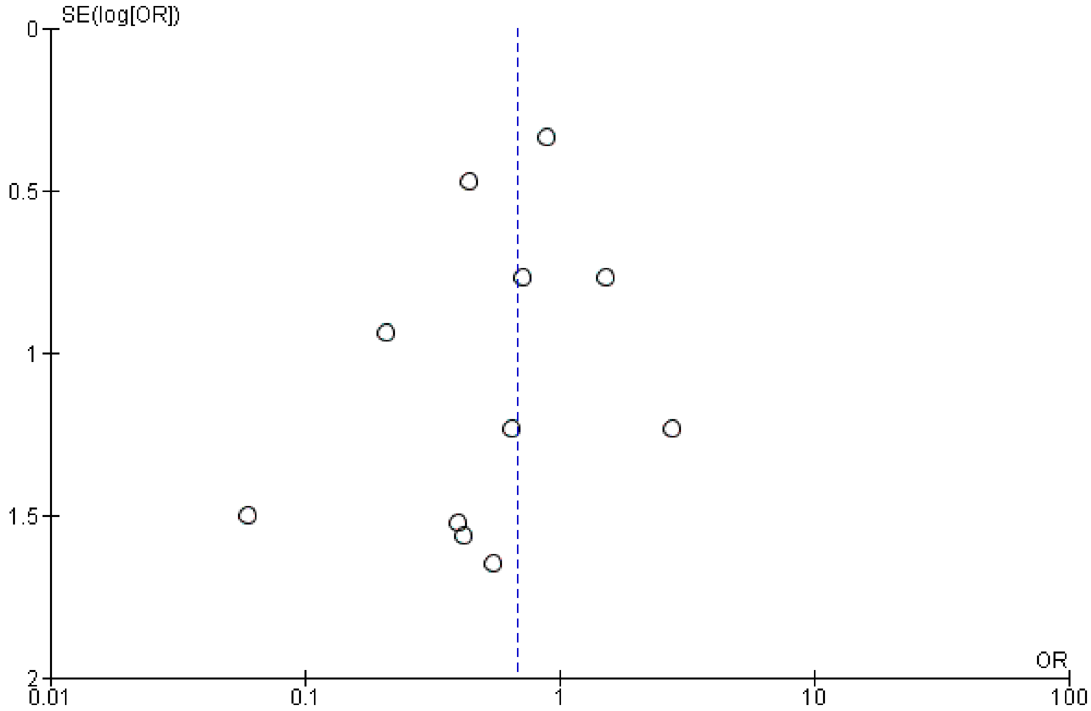

c. Funnel plot regarding cerebrovascular accident.

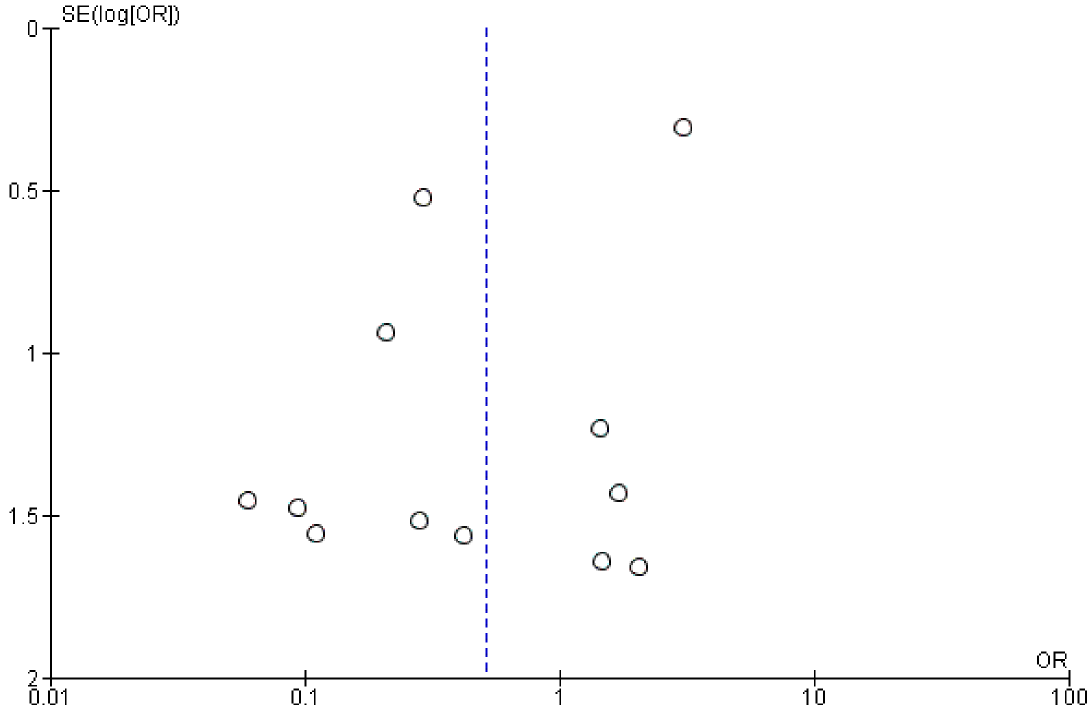

d. Funnel plot regarding conversion to sternotomy.

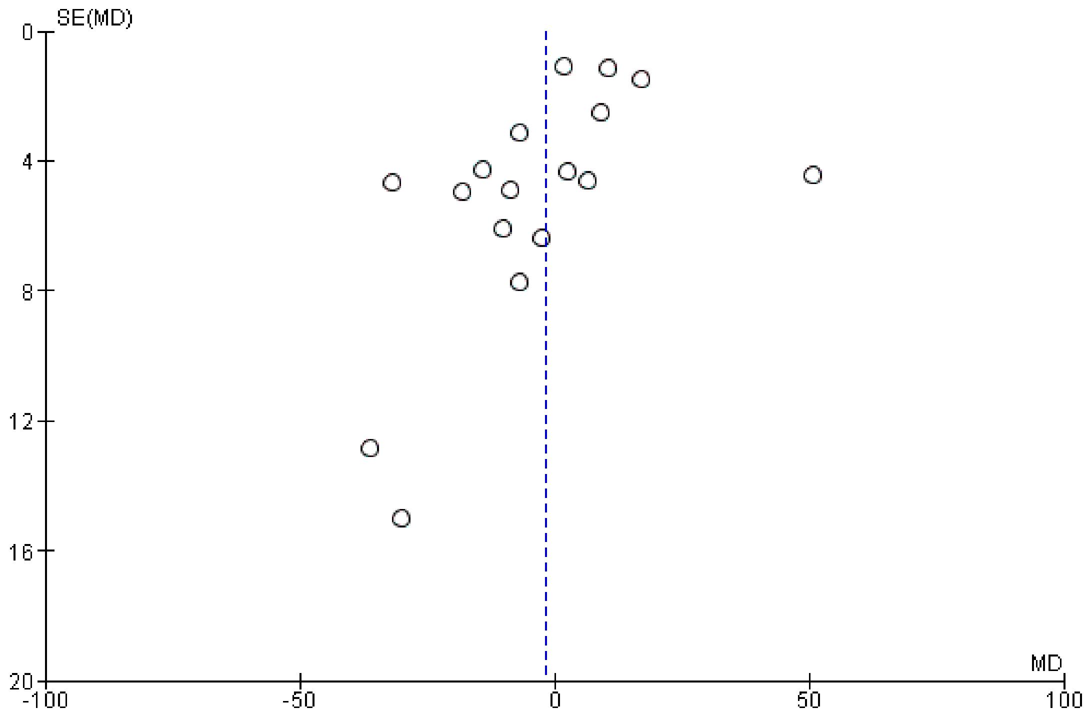

e. Funnel plot regarding cardiopulmonary bypass.

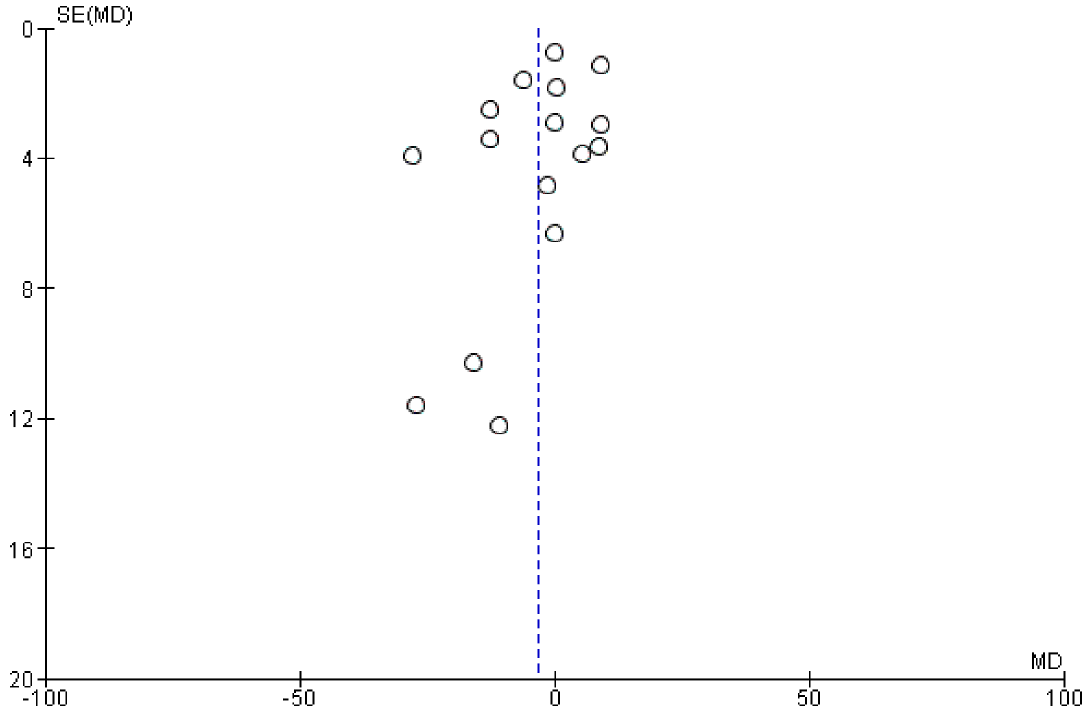

f. Funnel plot regarding aortic cross-clamp time.

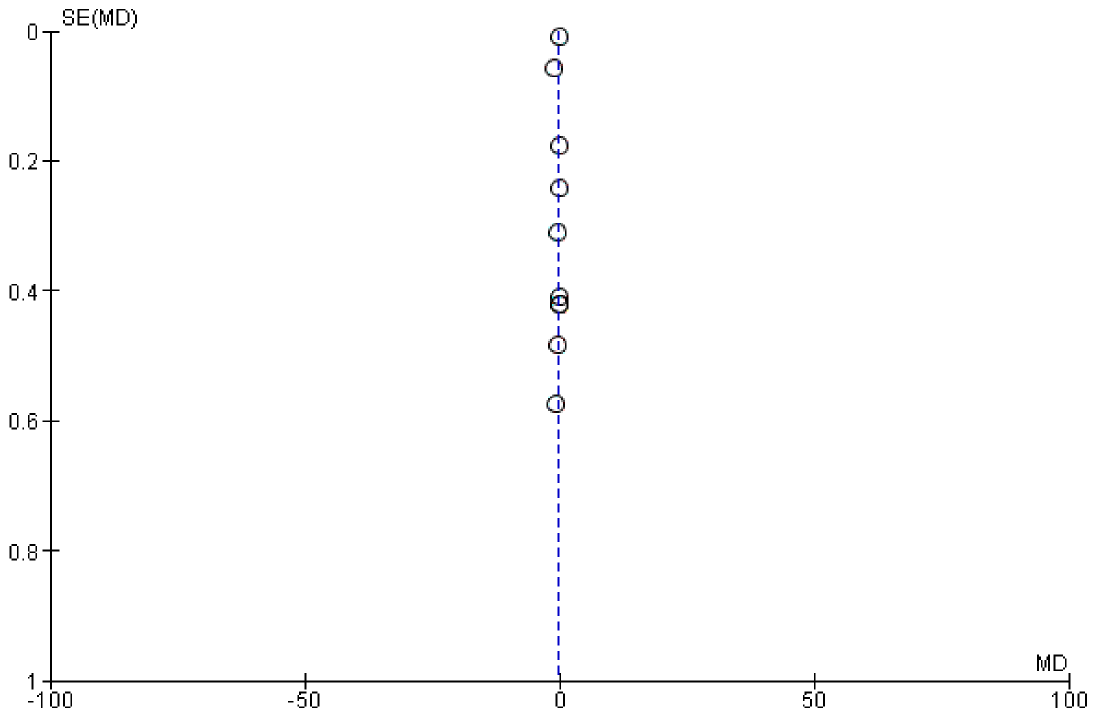

g. Funnel plot regarding intensive care unit stay.

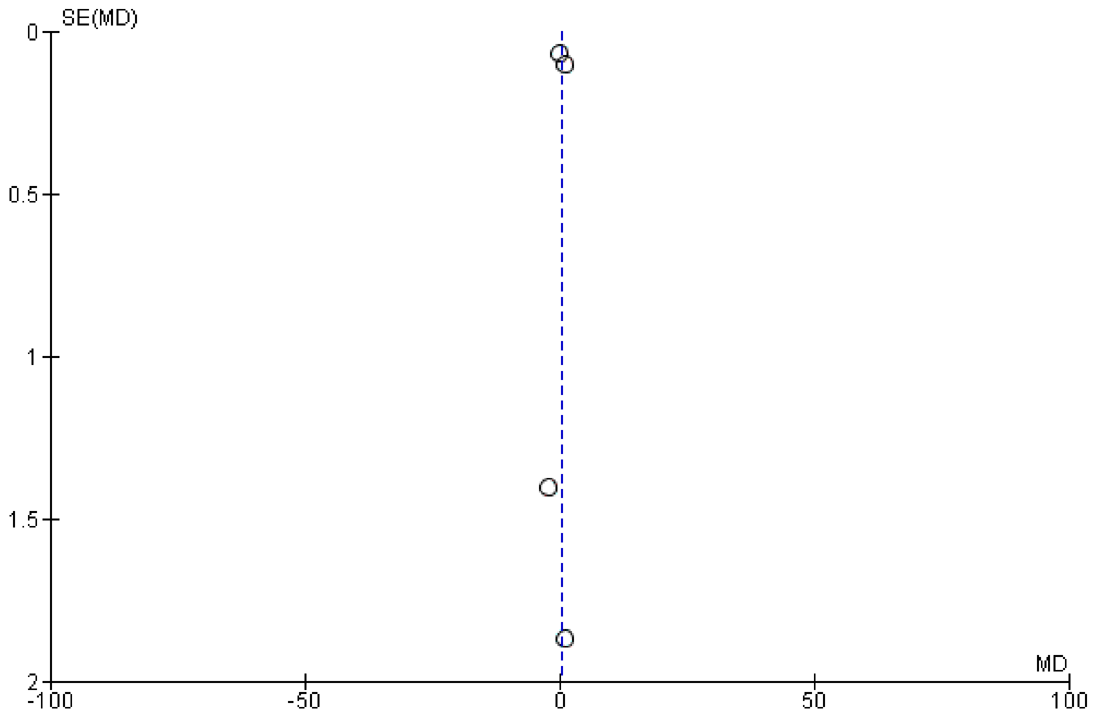

h. Funnel plot regarding length of stay.

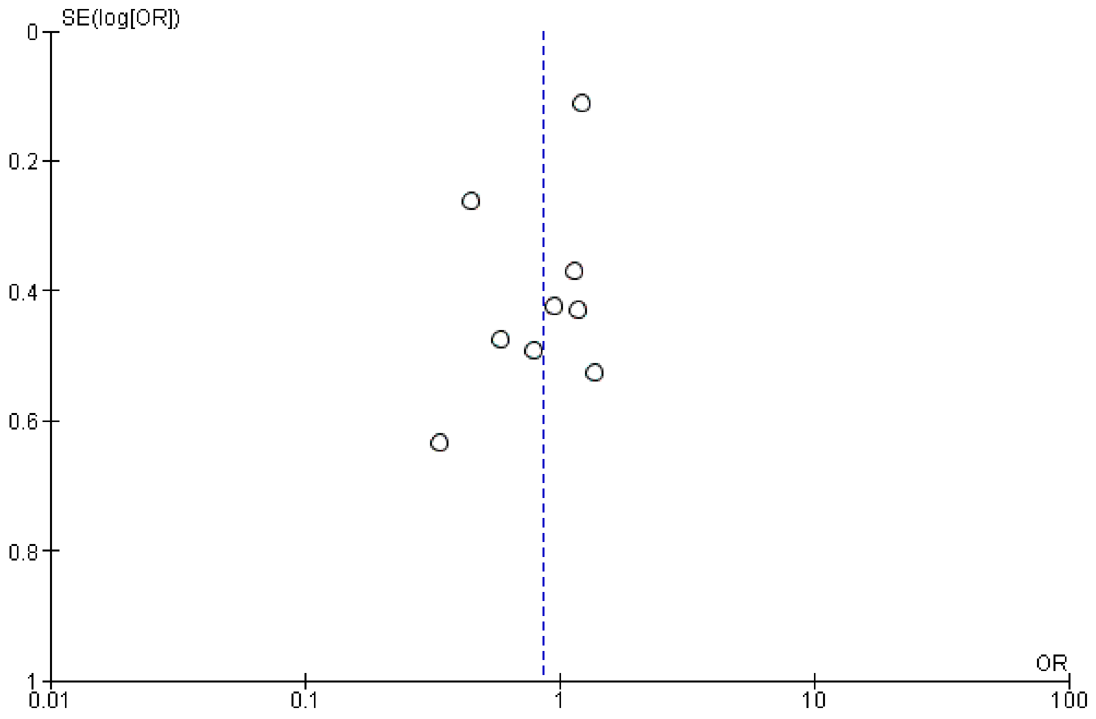

i. Funnel plot regarding postoperative atrial fibrillation.

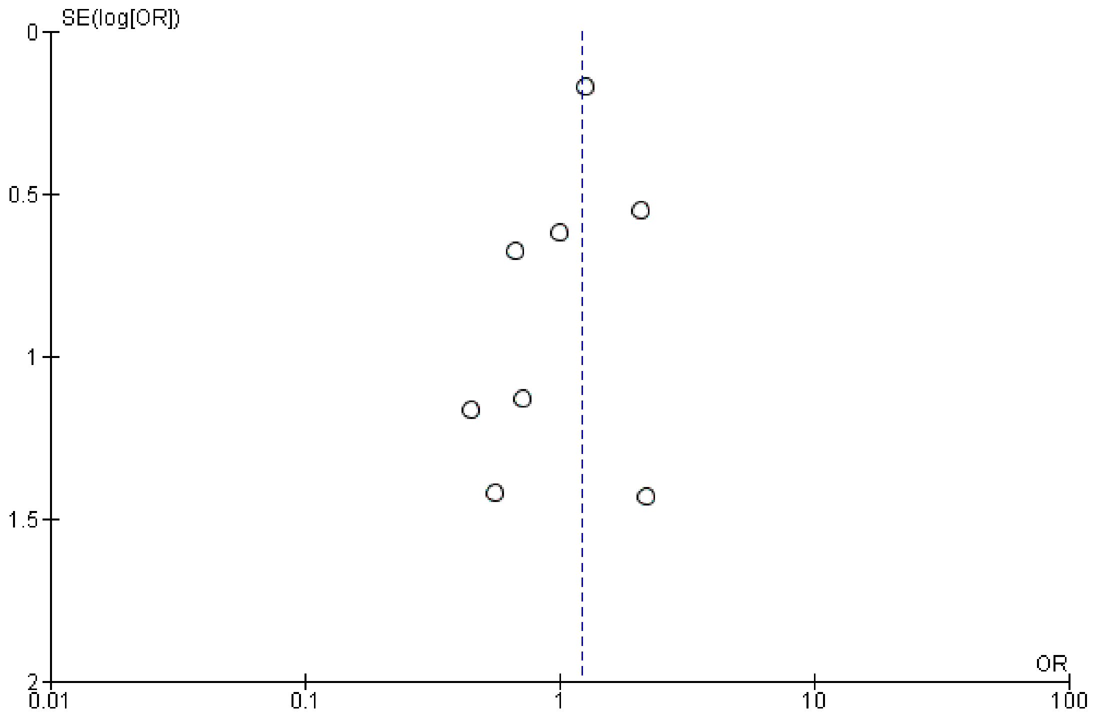

j. Funnel plot regarding acute kidney disease.

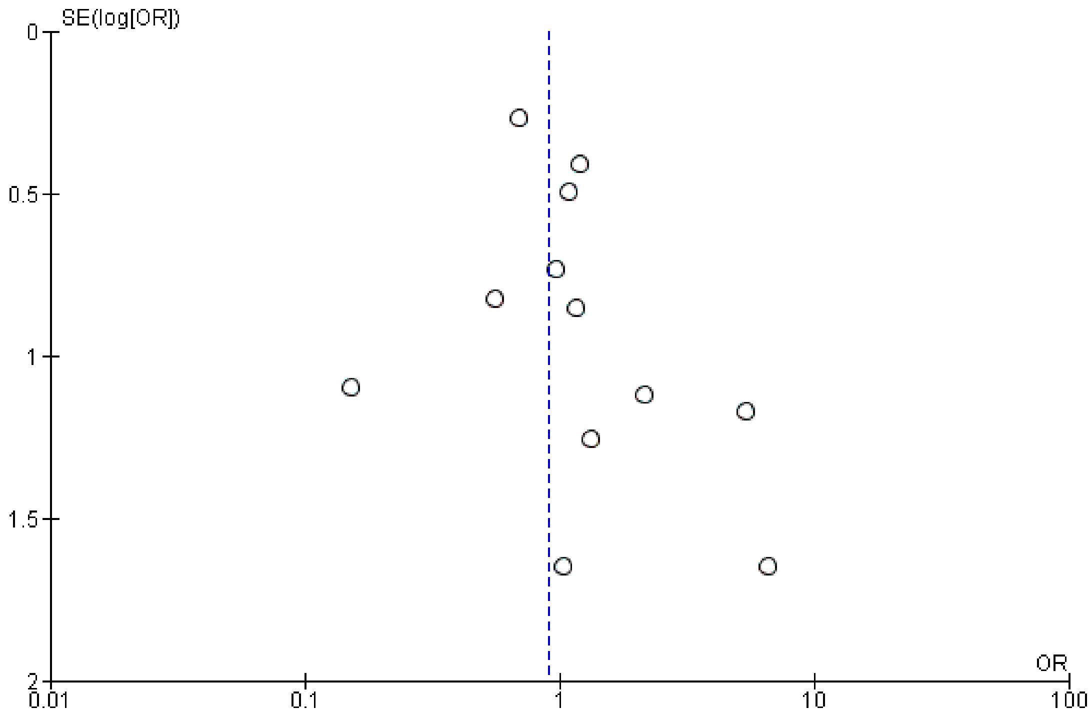

k. Funnel plot regarding re-exploration.

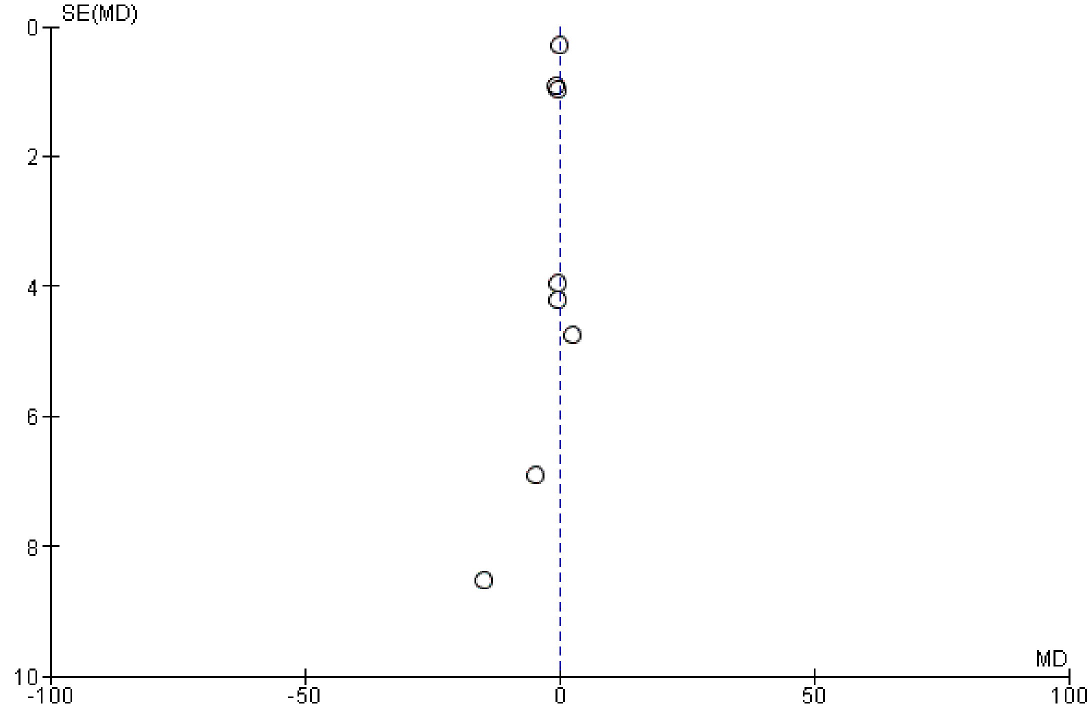

l. Funnel plot regarding ventilation.
